# Supplementary material for: Determinants of severe maternal outcome in Keren hospital, Eritrea: An unmatched case-control study
Source: PLoS One. 2024 May 13;19(5):e0299663. doi: 10.1371/journal.pone.0299663 (PMC11090357; doi:10.1371/journal.pone.0299663)
Supplement: Attachment — Submitted filename: Response to Reviewers.docx [file pone.0299663.s003.docx]

Dear Dr. Mergan Naidoo,

Thank you for giving me the opportunity to submit a revised version of the manuscript “*Determinants of severe maternal outcome in Keren hospital, Eritrea: an unmatched case-control study*” (Manuscript ID: PONE-D-22-28527R1) for publication in PLOS ONE. I have made necessary changes to the manuscript according to the suggestions provided during the review process. These changes are highlighted using tracked changes within the manuscript. Please see below for a point-by-point response to the comments. All page and line numbers refer to the **revised manuscript file with track changes**.

Thank you for considering this submission and I look forward to hearing from you.

**Point-by-point response to Editor’s comments**

**Response to Editor’s comments:**

1. **The introduction speaks to maternal near miss and its association with SMO but the study is not about MNM. This point has been previously pointed out. Please align the introduction with the research question.**

**Response:** Thank you for reminding me that the introduction section needs further amendments. I have rewritten major portion of the introduction section in the revised version of the manuscript. Now, the introduction mainly discusses the background on the determinants of maternal mortality and morbidity and on the aim of the study in relation to the context of previous studies. I have also made similar amendments in the “Background” sub-section of the “Abstract” section. I believe that the introduction section is now in tandem with the title and content of the manuscript. However, if there are additional issues that need to be addressed please let me know.

2. **It would also help if you included the SMOs and comorbidities for the cases and controls.**

**Response:** In this study, women who experienced severe maternal outcome (SMO) were considered as cases (i.e. women who encountered one or more organ dysfunction and women who died). Women who delivered without serious obstetric complications, on the other hand, were enrolled as controls. Hence, only women with SMO (cases) can be categorized as maternal deaths and maternal near misses. I have indicated in the results section that there were 662 near misses and 39 maternal deaths.

3. **Use the same fonts throughout the manuscript.**

**Response:** Thank you. In light of this comment, I have carefully assessed the entire manuscript for consistency of fonts. The font, “*Times New Roman*”, is used consistently throughout the manuscript. Moreover, font size “12” is applied for the entire text, except for the headings which are formatted in accordance with the PLoS ONE’s “Manuscript body formatting guidelines”.

4. **Capitalise words as appropriate i.e. proper nouns and when starting a sentence**

**Response:** Thank you for this comment. There were some instances where capitalization was not properly use in the former manuscript. Necessary corrections have now been made in the revised manuscript.

5. **Don't start a sentence with a number.**

**Response:** After careful revision, I have paraphrased all such sentences to avoid starting a sentence with a number.

6. **The use of bold text in the manuscript**

**Response:** again, thank you. In accordance with this comment, the revised manuscript does not contain any bold text with in the main body except for headings.

**Response to journal requirements:**

1. **Please review your reference list to ensure that it is complete and correct. If you have cited papers that have been retracted, please include the rationale for doing so in the manuscript text, or remove these references and replace them with relevant current references. Any changes to the reference list should be mentioned in the rebuttal letter that accompanies your revised manuscript. If you need to cite a retracted article, indicate the article’s retracted status in the References list and also include a citation and full reference for the retraction notice.**

**Response:** I have checked each and every reference in the manuscript, and I assure you that there is no any retracted paper cited in the reference list. I have made a number of changes to the reference list based on the comments given in the review process. I have indicated all changes made to the reference list in the **revised manuscript file with track changes** (including references removed from and added to the revised manuscript). In addition**,** I have added the doi for all peer-reviewed articles in the reference list. I have also added a link for the remaining references along with the date when they were accessed.
