# Supplementary material for: Determinants of severe maternal outcome in Keren hospital, Eritrea: An unmatched case-control study
Source: PLoS One. 2024 May 13;19(5):e0299663. doi: 10.1371/journal.pone.0299663 (PMC11090357; doi:10.1371/journal.pone.0299663)
Supplement: Attachment — Submitted filename: Response to Reviewers.docx [file pone.0299663.s004.docx]

Dear Dr. Mergan Naidoo,

Thank you for giving me the opportunity to submit a revised version of the manuscript “*Determinants of severe maternal outcome in Keren hospital, Eritrea: an unmatched case-control study*” (Manuscript ID: PONE-D-22-28527R2) for publication in PLOS ONE. I have made necessary changes to the manuscript according to the suggestions provided during the review process. These changes are highlighted using tracked changes within the manuscript. Please see below for a point-by-point response to the comments. All page and line numbers refer to the **revised manuscript file with track changes**.

Thank you for considering this submission and I look forward to hearing from you.

**Point-by-point response to Editor’s comments**

**Response to Editor’s comments:**

1. **In the abstract your conclusion must be aligned to your aim.**

**Response:** In accordance with the editor’s comments, I have rewritten the conclusion part of the abstract section. Now, the conclusion focuses on the main question the manuscript tries to address and the key recommendations for improvement.

2. **Please improve the quality of the academic writing by editing the manuscript. Some of the issues identified include: Not capitalizing proper nouns, using a number to start a sentence and the grammar.**

**Response:** Thank you for this comment. There were some instances where capitalization was not properly used in the former manuscript. Necessary corrections have now been made in the revised manuscript. I have also carefully revised the manuscript to make sure that there are no sentences that start with a number. Moreover, all necessary amendments were made to avoid grammatical mistakes in the revised manuscript. If you still think that there are unresolved issues in this area, please let me know. It would also be helpful if you could mention one or two examples where such errors remain uncorrected.

3. **Please ensure that the references comply with the Vancouver referencing format especially when using online databases and websites.**

**Response:** I have carefully studied the guide for the Vancouver referencing style and I have made minor adjustments to the reference list to make sure that it adheres with the recommended format.

**Response to journal requirements:**

1. **Please review your reference list to ensure that it is complete and correct. If you have cited papers that have been retracted, please include the rationale for doing so in the manuscript text, or remove these references and replace them with relevant current references. Any changes to the reference list should be mentioned in the rebuttal letter that accompanies your revised manuscript. If you need to cite a retracted article, indicate the article’s retracted status in the References list and also include a citation and full reference for the retraction notice.**

**Response:** I have checked each and every reference in the manuscript, and I assure you that there is no any retracted paper cited in the reference list. I have indicated in this rebuttal letter all the changes made to the reference list.
